# AhR Ligands Modulate the Differentiation of Innate Lymphoid Cells and T Helper Cell Subsets That Control the Severity of a Pulmonary Fungal Infection

**DOI:** 10.3389/fimmu.2021.630938

**Published:** 2021-04-16

**Authors:** Eliseu F. de Araújo, Flávio V. Loures, Nycolas W. Preite, Cláudia Feriotti, Nayane AL Galdino, Tânia A. Costa, Vera L. G. Calich

**Affiliations:** Department of Immunology, Institute of Biomedical Sciences, University of São Paulo, São Paulo, Brazil

**Keywords:** innate lymphoid cells (ILCs), L-kynurenine, FICZ, T cell subsets, paracoccidiodomycosis, IDO - Indoleamine 2 3-dioxygenase, AhR (Aryl hydrocarbon Receptor)

## Abstract

In agreement with other fungal infections, immunoprotection in pulmonary paracoccidioidomycosis (PCM) is mediated by Th1/Th17 cells whereas disease progression by prevalent Th2/Th9 immunity. Treg cells play a dual role, suppressing immunity but also controlling excessive tissue inflammation. Our recent studies have demonstrated that the enzyme indoleamine 2,3 dioxygenase (IDO) and the transcription factor aryl hydrocarbon receptor (AhR) play an important role in the immunoregulation of PCM. To further evaluate the immunomodulatory activity of AhR in this fungal infection, *Paracoccidioides brasiliensis* infected mice were treated with two different AhR agonists, L-Kynurenin (L-Kyn) or 6-formylindole [3,2-b] carbazole (FICZ), and one AhR specific antagonist (CH223191). The disease severity and immune response of treated and untreated mice were assessed 96 hours and 2 weeks after infection. Some similar effects on host response were shared by FICZ and L-Kyn, such as the reduced fungal loads, decreased numbers of CD11c+ lung myeloid cells expressing activation markers (IA, CD40, CD80, CD86), and early increased expression of IDO and AhR. In contrast, the AhR antagonist CH223191 induced increased fungal loads, increased number of pulmonary CD11c+ leukocytes expressing activation markers, and a reduction in AhR and IDO production. While FICZ treatment promoted large increases in ILC3, L-Kyn and CH223191 significantly reduced this cell population. Each of these AhR ligands induced a characteristic adaptive immunity. The large expansion of FICZ-induced myeloid, lymphoid, and plasmacytoid dendritic cells (DCs) led to the increased expansion of all CD4+ T cell subpopulations (Th1, Th2, Th17, Th22, and Treg), but with a clear predominance of Th17 and Th22 subsets. On the other hand, L-Kyn, that preferentially activated plasmacytoid DCs, reduced Th1/Th22 development but caused a robust expansion of Treg cells. The AhR antagonist CH223191 induced a preferential expansion of myeloid DCs, reduced the number of Th1, Th22, and Treg cells, but increased Th17 differentiation. In conclusion, the present study showed that the pathogen loads and the immune response in pulmonary PCM can be modulated by AhR ligands. However, further studies are needed to define the possible use of these compounds as adjuvant therapy for this fungal infection.

## Introduction

The aryl hydrocarbon receptor (Ahr), a ligand-dependent transcription factor that resides in the cytoplasm of many cell types, was first described due to its involvement in the metabolism of xenobiotic compounds such as dioxin (1). Currently, it is well known that AhR is activated by a diverse set of endogenous and exogenous ligands (2). At a steady-state, AhR remains in the cytoplasm (3) but translocates to the nucleus after ligand binding. In the nucleus, AhR heterodimerizes with AhR Nuclear Translocator (ARNT) and then interacts with its genomic binding motifs inducing the transcription of its target genes, including detoxifying enzymes of the cytochrome P_450_ family (4). AhR also interacts with other transcription factors that regulate AhR signaling (3). It was also reported that the ligand structure and affinity control AhR activity (5). Several AhR ligands were described: L-Kynurenines (L-Kyn), products of tryptophan degradation by the enzymatic action of indoleamine 2,3 dioxygenase (IDO), 6-formylindole [3,2-b] carbazole (FICZ), a tryptophan photoproduct, and several microbial and dietary products (5–7). AhR is expressed by innate and adaptive immune cells and influences the development and activation of the immune system. This transcription factor plays an important role in the control of cell differentiation, proliferation, and cytokines production (7–11). Indeed, AhR was shown to exert an important activity on T helper 17 (Th17) and regulatory T cells (Treg) differentiation, influencing the severity of several experimental pathologies (9–11). Innate lymphoid cells (ILCs), a family of immune cells that do not express antigen receptors but exhibit phenotypes that reflect Th cell subpopulations, were also reported to be regulated by the AhR expression. The differentiation of ILC3 and lymphoid tissue inducing lymphocyte (LTi), that secrete IL-17, IL-22, and lymphotoxin is dependent on the transcription factors RORγτ and AhR (12).

The regulatory activity of AhR has been demonstrated in several infectious pathologies (13). However, the effects of AhR activation were not homogeneous due to the various AhR ligand used, type of pathology studied, and treatment protocols employed (3, 13–15).

*P. brasiliensis*, a fungal pathogen endemic to Latin America, is sensed by a variety of pattern recognition receptors that stimulate the differentiation of a wide range of T cell subpopulations involved in host immunity (16–23). In humans and experimental models of PCM (PCM), Th1/Th17 promote immunoprotection: Th1 by controlling fungal loads *via* IFN-γ activated macrophages and Th17 by promoting neutrophil recruitment and activation. Th2 and Th9 cells are associated with increased fungal growth, inefficient inflammatory reactions, and disease severity (18, 19, 24, 25). In the human disease, Treg cells are associated with the progressive and severe forms of the disease (25–28), but experimental models of pulmonary PCM clearly showed the dual role of this T cell subset: it is deleterious due to its suppressive effect on protective immunity but it has also a beneficial effect mediated by the inhibition of excessive inflammatory reactions (24, 28, 29).

In experimental candidiasis, the enzyme indoleamine 2,3 dioxygenase (IDO), which regulates tryptophan (Trp) degradation, was shown to reduce fungal loads but also to control immunity by reducing Th17 expansion *via* increased Treg cell proliferation mediated by L-Kyn-activated AhR (30–32). Moreover, AhR was also involved in the protection of *Candida albicans* infected mucosae (32) due to its regulatory activity on IL-22 production (9, 33). In pulmonary PCM, our recent studies have shown that *P. brasiliensis* infection induces a vigorous IDO expression that mediates Trp catabolism, resulting in increased L-Kyn production and AhR activation. *P. brasiliensis* uses two distinct mechanisms to trigger IDO expression. In susceptible (B10.A) mice, IDO is induced by IFN-γ and exhibits a prevalent enzymatic activity whereas in resistant (A/J) mice IDO is TGF-*β* induced and behaves as a signaling molecule (34–36). Our studies have also demonstrated that IDO and AhR are mutually regulated and control the number of ILCs and the Th17/Treg balance (34–37). Altogether, our findings defined the important regulatory role of the IDO/AhR axis in the immunity and severity of pulmonary PCM leading us to better evaluate the role of AhR in pulmonary PCM. To this aim, *P. brasiliensis* infected mice were treated with three different AhR ligands, two agonists (L-Kyn and FICZ) and an antagonist (CH223191), and disease severity and immune response assessed 96 hours and 2 weeks after infection. We verified that AhR ligands control fungal burdens, cytokines production, and activation of pulmonary myeloid cells. Importantly, FICZ showed a prevalent effect on the differentiation of Th17 and Th22 cells, L-Kyn on Tregs, and CH223191 on Th17 cells. Altogether, our findings demonstrate that pulmonary PCM can be modulated by AhR ligands that could be used to regulate the differentiation of pro- or anti-inflammatory T cell subsets.

## Materials and Methods

### Ethics Statement

The experiments were performed in strict accordance with the Brazilian Federal Law 11,794 establishing procedures for the scientific use of animals, and the State Law establishing the Animal Protection Code of the State of São Paulo. All efforts were made to minimize animal suffering. The procedures were approved by the Ethics Committee on Animal Experiments of the Institute of Biomedical Sciences of the University of São Paulo (Proc.180/11/CEEA).

### Mice

C57B/6 SPF male mice, bred at the Isogenic Breeding Unit of the Department of Immunology, Institute of Biomedical Sciences, were used at the age of 6-8 weeks.

### Fungus and Intratracheal (i.t.) Infection

The virulent Pb18 isolate from *P. brasiliensis* was maintained in the yeast form by weekly cultivation in Fava Netto’s semi-solid medium at 36°C and used on days 6–8 of culture. The fungus was collected and washed with phosphate-buffered saline (PBS, pH 7.2). The fungal viability was determined by the Janus Green B vital dye. All experimental procedures were carried out with fungal suspensions presenting viability between 90 and 95%. For i.t. infection, mice were anesthetized with ketamine and xylazine and submitted to i.t. infection with 1x10^6^ yeast cells, contained in 50 µL of PBS as previously described (35).

### Treatment of Mice With AhR Agonists and Antagonist

C57BL/6 mice were infected as described above and treated with two different AhR agonists, 6-formylindol [3,2-b] carbazole (FICZ, Enzo Labs) or L-Kynurenine (L-Kyn, Sigma Aldrich). The drug 2-methyl-2H-pyrazole-3-carboxylic acid-amide (CH223191-Signa Aldrich) was employed as an AhR antagonist. Stock solutions of L-Kyn (20 mg/ml, 96 mM), FICZ (2 mg/ml, 7 mM) and CH223191 (30 mg/ml, 90 mM) were prepared in DMSO. These drugs were properly diluted in phosphate buffered solution (PBS) just before use. After i.t. infection, mice were inoculated intraperitoneally on alternate days with 200 μg of FICZ, or 400 μg of CH223191or 800 μg of L-Kyn per animal, contained in 500 μl of diluent solution. PBS was used in control infected mice. These protocols were adapted from those previously described (38–41).

### Assessment of Disease Severity by CFU Counts

The disease severity of control and ligand-treated infected mice was assessed after 96 hours and 2 weeks of infection. The analysis was carried out by recovering viable fungal cells from lungs, liver, and spleen, using a BHI medium supplemented with horse serum and a culture filtrate obtained from *P. brasiliensis* (isolate 192).

### Preparation of Cell Suspensions

Lung cell suspensions were prepared as previously described (34). The lungs were removed and digested for 30 min. in digestion buffer containing collagenase (Sigma). The organs were then macerated in a homogenizer with RPMI 1640 culture medium. The erythrocytes were lysed with lysis buffer, the cells counted, and their viability assessed by Trypan blue dye.

### Flow Cytometry for Characterization of Cellular Subpopulations

Lung cell suspensions were adjusted to 1x10^6^ cells and suspended in PBS-azide (0.1%) containing fetal bovine serum (SFB, 5%). Fc receptors were blocked with anti-CD16/32 monoclonal antibody and then labeled with fluorophore-conjugated antibodies as previously described (37) Labeled antibodies (BD Biosciences) were used in the appropriate combination for the cell population to be analyzed. For lymphocytes, the following antibodies were used: anti-CD3, CD4, CD25, and Foxp3; for myeloid cells: anti-CD45, CD11b, CD11c, CD40, CD80, CD86, MHC-II, and F4/80. For ILCs characterization, lung leukocytes were first treated with an anti-mouse lineage cocktail (Biolegend) containing antibodies to CD3, Ly6G/Ly6C, CD11b, CD45R/B220, TER 119/erythroid cells, that react with T cells, B cells, monocytes, macrophages, NK cells, and erythrocytes. Intracellular staining was conducted using the eBioscience Transcription Factor staining kit and specific antibodies for IL-17, IL-4, IFN-γ, IL-22, IL-1β, IL-12, TNF-α, IL-6, TGF-β, IL-10, FoxP3, IDO-1, and AhR. **Supplementary Table 1** lists the monoclonal antibodies used in flow cytometry assays. Cells were run on FACSCantoII (BD Biosciences) and a minimum of 50,000 events was acquired using FACSDiva software (BD Biosciences). Cells were analyzed using FlowJo software (Tree Star).

### Cytokines Detection (ELISA)

The presence and concentration of cytokines (IL-12, TNF-α, IFN-γ, IL-1β, IL-4, IL-10, TGF-β, IL-35, IL-6, IL-23, IL-17, and IL-22 were determined in lung homogenates obtained 96 h and 2 weeks after infection of AhR ligands treated and untreated mice. The methodology used was that recommended by the supplier (EBioscience).

### Real-Time PCR (qPCR)

RNA isolation from lung macerates of AhR ligand-treated and untreated mice was performed as previously described (37). A NanoDrop ND-1000 spectrophotometer was used to determine RNA purity and concentration. The cDNA was synthesized using 1 µg of RNA and the high-capacity RNA-to-cDNA kit (Applied Biosystems) according to the manufacturer’s instructions. The cDNA was amplified using TaqMan Universal PCR Master Mix (Applied Biosystems) and pre-developed TaqMan assay primers and probes (*Ifng*, Mm001168134_m1, *Tnf*, Mm99999068_m1, *Il6*, Mm00446190_m1, *Il10*, Mm00439614_m1, *Tgfb1*, Mm00117882_m1, *Il17*, Mm00439618_m1, *Il22*, Mm01226722_m1, *Tbet*, Mm00450960_m1; *Gata3*, Mm00484683_m1; *Rorc*, Mm01261022_m1; *Foxp3*, Mm00475162_m1; *Gapdh*, Mm99999915_g1a, all from Applied Biosystems.). PCR assays were performed on an MxP3000P QPCR System and data were developed using the MxPro qPCR software (Stratagene). The average threshold cycle (CT) values of samples were normalized to the CT value of the *Gapdh* gene. The relative expression was determined by the 2^-ΔΔ^CT method.

### Statistical Analysis

Data were analyzed as previously described (42) and expressed as the M ± SD. Differences between groups were tested using a one-way analysis of variance (ANOVA) followed by the Dunnet’s *post hoc* test to compare every mean with a control mean. Data were analyzed using GraphPad Prism 7.03 software (GraphPad Prism Software, Inc.). A *P* value ≤ 0.05 was considered significant.

## Results

### Treatment With AhR Agonists (FICZ and L-Kyn) Reduces, While the Antagonist (CH223191) Increases the Pulmonary Fungal Load of *P. brasiliensis* Infected Mice

**Figure 1:**
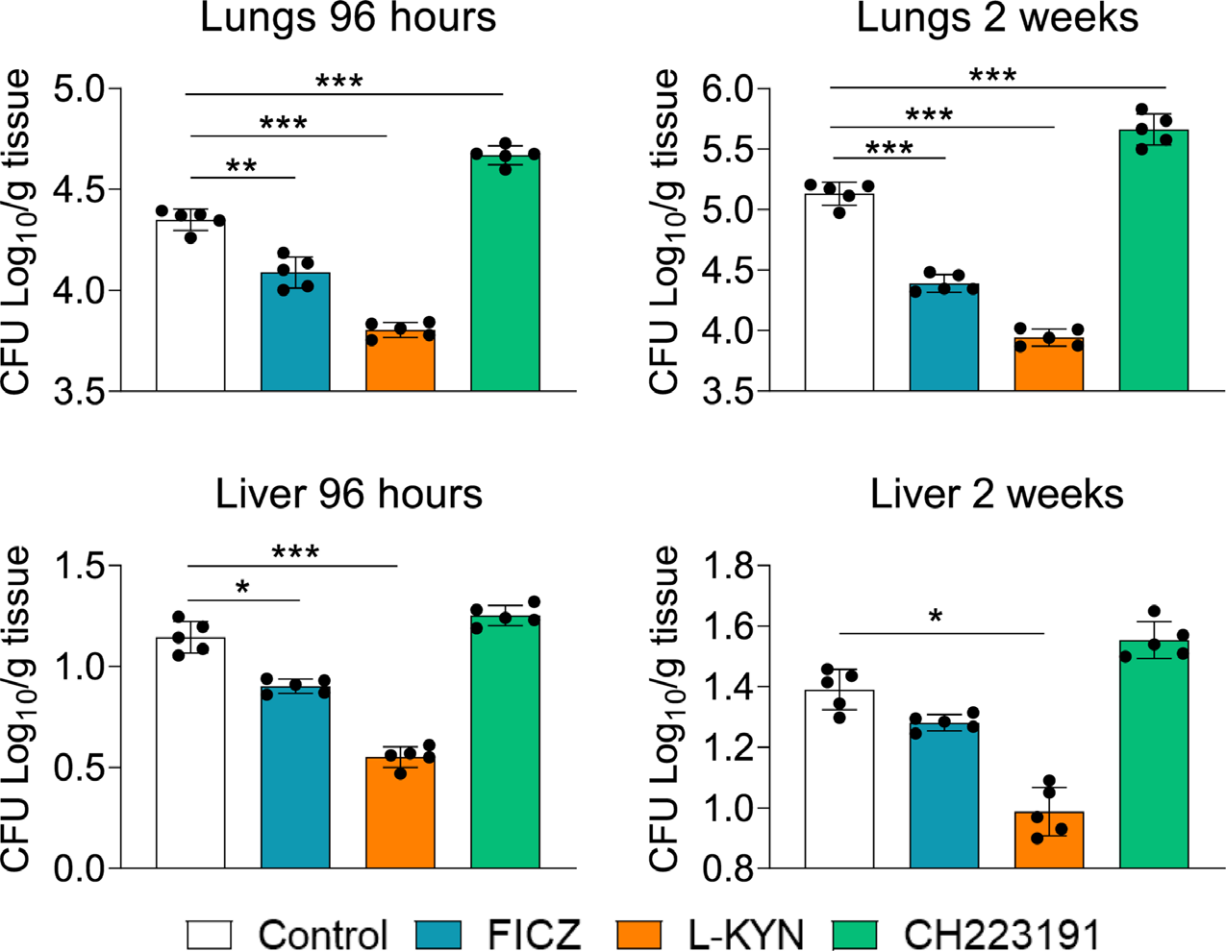
Treatment with AhR agonists (FICZ and L-Kyn) reduces while the antagonist (CH223191) increases the fungal load in mice infected with *P. brasiliensis*. C57BL/6 mice (n = 5) were infected with 1x10^6^ yeasts of *P. brasiliensis* and treated by route i,p. on alternate days with FICZ, L-Kyn, or CH22391 at doses of 200 μg or 800 μg or 400 μg/animal, respectively. Control mice were treated with the PBS. The animals were sacrificed 96 hours and 2 weeks after infection, their lungs and liver removed, macerated, and evaluated for the fungal load. The experiment was repeated twice, and data are expressed as M ± SD. *P* values < 0.05 were considered significant (*p < 0.05; **p < 0.005 and ***p < 0.001).

C57BL/6 male mice were infected with 1 x 10^6^ *P. brasiliensis* yeasts and groups of 5 animals were treated with the AhR agonists L-Kyn (800 μg i.p./mice) or FICZ (200 μg i.p./mice) every other day starting at day-1 of fungal infection. Another group was treated with the AhR antagonist CH223191 (400 μg i.p./mice) on alternate days after infection. Control mice were infected and treated with the drug vehicle following the same protocol above described. The AhR ligands treated and untreated infected mice were sacrificed 96 hours and 2 weeks after infection, their lungs and liver macerated, and the presence of viable fungi evaluated by the colony-forming units (CFU) method. **Figure 1** shows that there was a significant reduction in pulmonary and hepatic fungal loads in FICZ and L-Kyn treated mice at both assayed periods; in contrast, treatment with CH223191 increased the fungal load of the lungs, but not that of the liver.

### Treatment With AhR Agonists (FICZ and L-Kyn) Decreases While the Antagonist CH223191 Increases the Number of Activated Myeloid Cells (CD11c+) in *P. brasiliensis* Infected Mice

**Figure 2:**
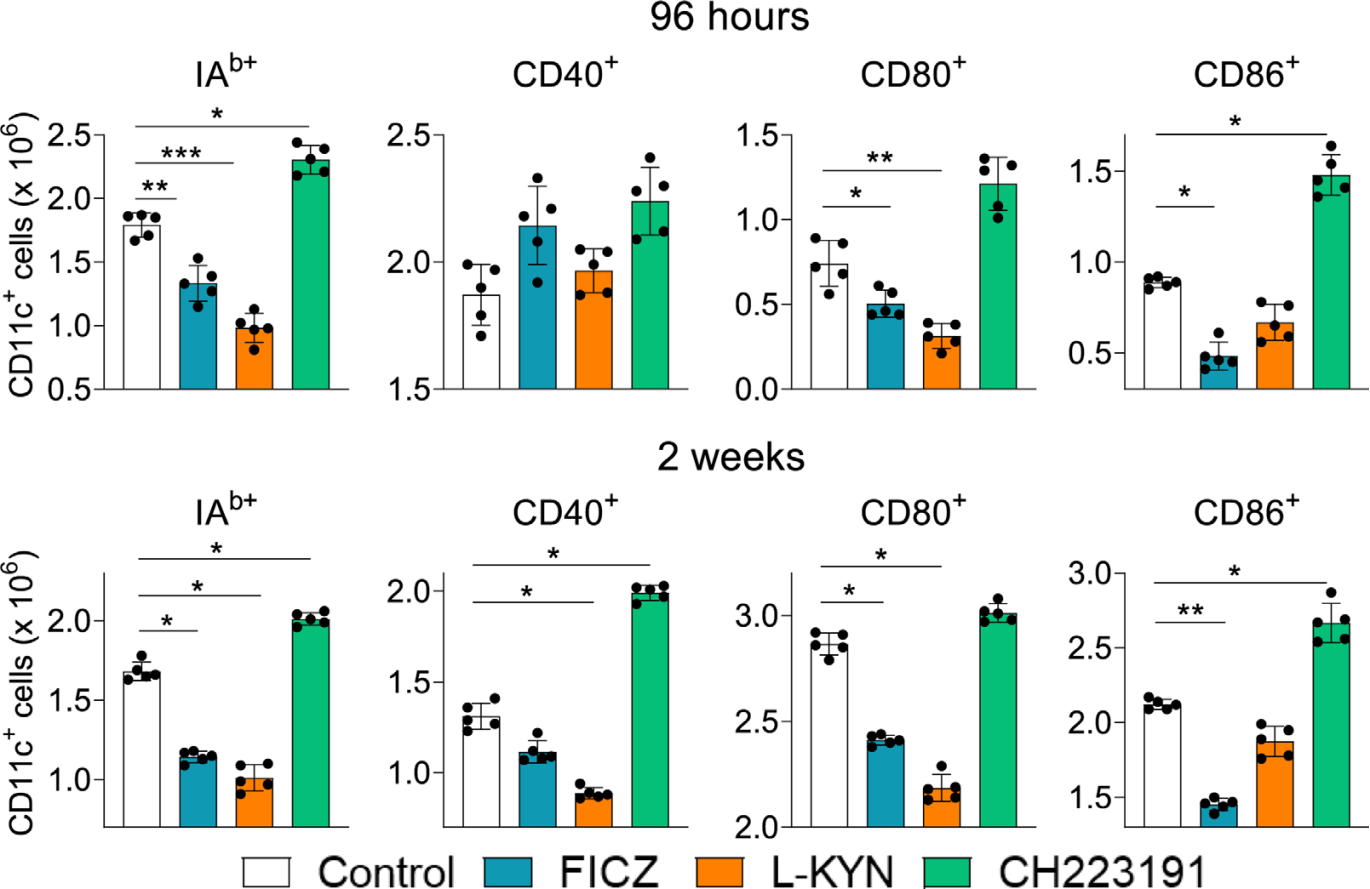
Treatment with AhR agonists (FICZ and L-Kyn) reduces, while the antagonist (CH223191) increases the number of activated myeloid CD11c+ cells in the lungs of *P. brasiliensis* infected mice. C57BL/6 mice (n = 5) were infected with 1 x 10^6^ *P. brasiliensis* yeasts and treated by route i,p. on alternate days with FICZ, L-Kyn, or CH22 according to the protocol previously described. Control mice were treated with PBS. The animals were sacrificed 96 hours and 2 weeks after infection, their lungs removed, macerated, and CD11c+ leukocytes analyzed by flow cytometry for the expression of activation markers (IA^b^, CD40, CD80, and CD86). The experiment was repeated twice, and data are expressed as M ± SD. *P* values < 0.05 were considered significant (*p < 0.05; **p < 0.005 and ***p < 0.001).

Treated and untreated infected mice were sacrificed 96 hours and 2 weeks after infection, their lungs removed, macerated and CD11c+ lung myeloid cells analyzed by flow cytometry for the expression of activation markers (IA^b^, CD40, CD80, and CD86). As can be seen in **Figure 2**, the number of activated CD11c+ myeloid cells was present in reduced numbers in the lungs of mice treated with AhR agonists. In contrast, treatment with the CH223191 antagonist increased the number of activated CD11c+ cells in the lungs of infected mice.

### Treatment With AhR Ligands Alters the Intracellular Expression of IDO, AhR, and Cytokines by CD11c+ Myeloid Cells

**Figure 3:**
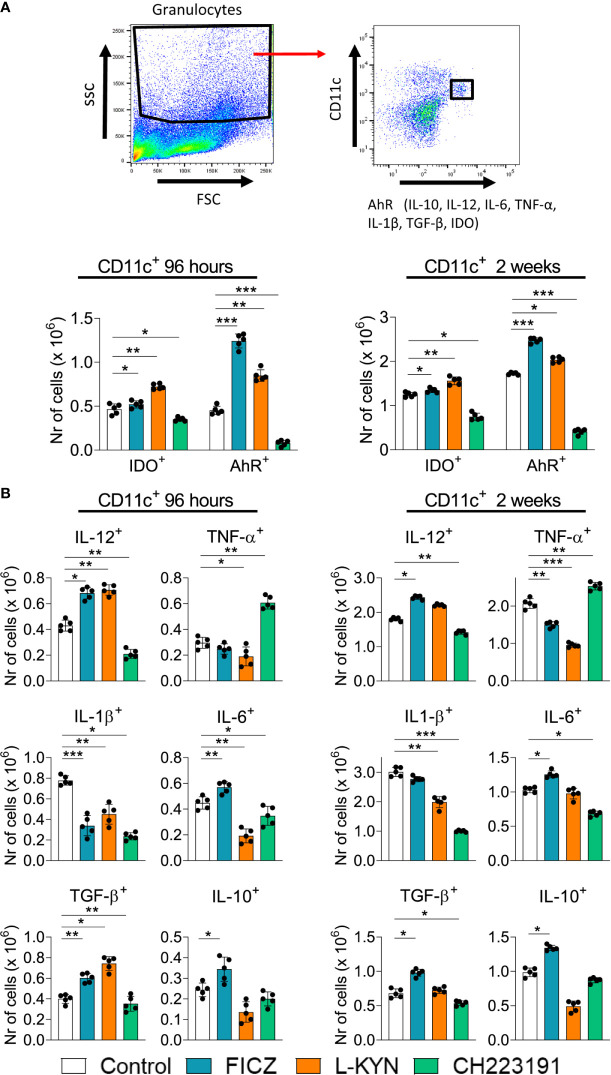
Treatment with AhR ligands (FICZ, L-Kyn, and CH2231991) alters the intracellular expression of IDO, AhR, and cytokines by pulmonary myeloid CD11c+ cells from *P. brasiliensis* infected mice. C57BL/6 mice (n = 5) were infected with 1 x 10^6^ *P. brasiliensis* yeasts and treated by route i,p. on alternate days with FICZ, L-Kyn, or CH223191 at doses of 200, 800, or 400 μg/animal, respectively. Control mice were treated with PBS. The animals were sacrificed 96 hours and 2 weeks after infection, their lungs removed, macerated and CD11c+ leukocytes analyzed by flow cytometry for the intracellular expression of the enzyme IDO, the AhR transcription factor, and cytokines (IL-12, TNF-α, IL -1β, IL-6, TGB-β and IL-10). **(A)** Gate strategy to define CD11c+ expressing IDO, AhR, and cytokines. **(B)** Number of pulmonary CD11c+ cells expressing AhR, IDO and cytokines detected at 96 hours and 2 weeks after infection. The experiment was repeated twice, and data are expressed as M ± SD. *P* values < 0.05 were considered significant (* *p < *0.05; **p < 0.005 and ***p < 0.001).

Mice were infected and treated as above described. The animals were sacrificed 96 hours and 2 weeks after infection, their lungs removed, macerated and the leukocytes analyzed by flow cytometry for the intracellular expression of IDO, AhR, and cytokines (IL-12, TNF-α, IL-1β, IL-6, TGB-β, and IL-10) by CD11c+ myeloid cells. **Figure 3A** shows the gating strategy used to characterize these cells. As can be seen in **Figure 3B**, at 96 hours and 2 weeks after infection both agonists increased while the antagonist reduced the number of CD11c+ cells expressing intracellular IDO and AhR. As for cytokine expression, a general view suggests that FICZ led to increased, while CH223191 to a reduced number of CD11c+ cells expressing intracellular cytokines. Interestingly, all treatments at both periods assayed caused a robust reduction in IL-1β+ CD11c+ cells. At both post-infection periods, FICZ increased the numbers of CD11c+ cells expressing IL-12, IL-6, TGF-β and IL-10. L-Kyn reduced the number of TNF-α+ and IL-1β+ CD11c+ cells at both infection periods but increased the number of IL-12 and TGF-β expressing CD11c+ cells by 96 hours of infection. On the other hand, treatment with CH223191increased the number of CD11c+ myeloid cells expressing TNF-α but reduced those producing IL-12, IL-1β, and IL-6 at both time points assayed.

### Treatment With AhR Ligands Increases the Migration of Dendritic Cells (DCs) to the Lungs of *P. brasiliensis* Infected Mice

**Figure 4:**
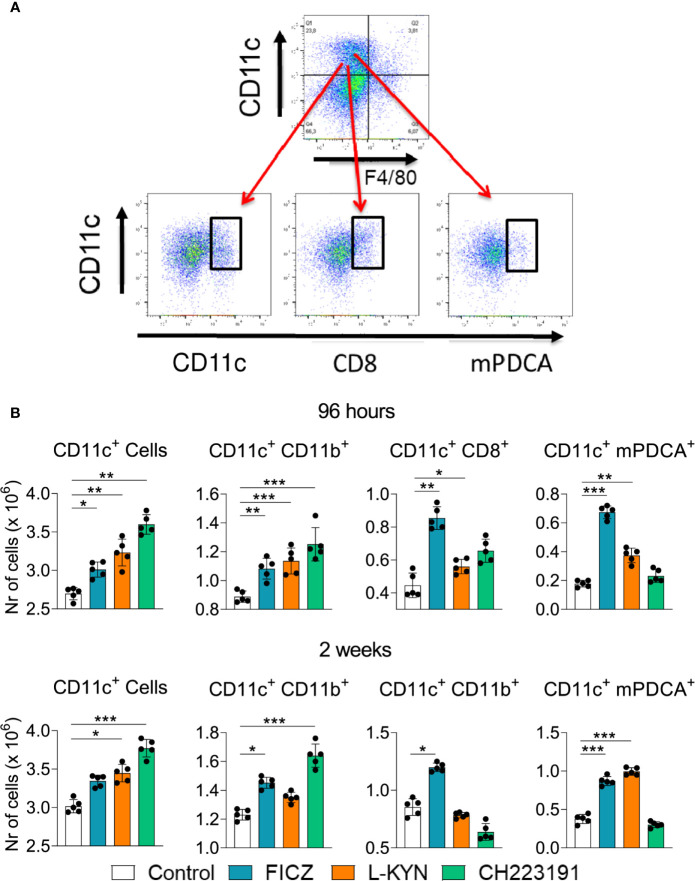
Treatment with AhR agonists (FICZ and L-Kyn) and antagonist (CH223191) increases the migration of dendritic cells (DCs) to the lungs of *P. brasiliensis* infected mice. C57BL/6 mice (n = 5) were infected with 1 x 10^6^ *P. brasiliensis* yeasts and treated by route i,p. on alternate days with FICZ, L-Kyn, or CH223191 as previously described. Control mice were treated with PBS. The animals were sacrificed 2 weeks after infection, their lungs removed, macerated and the number total CD11c+, myeloid (CD11c+CD11b +), lymphoid (CD11c+CD8+), and plasmacytoid (CD11c+mPDCA+) dendritic cells analyzed by flow cytometry. **(A)** Gate strategy to define DCs subsets. **(B)** Number of total pulmonary CD11c+ cells and DCs subsets observed 2 weeks after infection. The experiment was repeated twice, and data are expressed as M ± SD. *P* values < 0.05 were considered significant (* *p < *0.05; **p < 0.005 and ***p < 0.001).

Mice were treated as previously described and analyzed two weeks post-infection by flow cytometry regarding the presence of myeloid (CD11c+CD11b+), lymphoid (CD11c+CD8+), and plasmacytoid (CD11c+mPDCA+) DCs in the lungs of infected mice. **Figure 4A** shows the gating strategy used to define DCs subpopulations. The number of CD11c+ cells increased in the lungs of mice receiving all three treatments and at both time points assayed (**Figure 4B**). All DC subpopulations were found in higher numbers in FICZ treated mice at both post-infection periods. L-Kyn also increased all DC subsets by 96 hours after infection but only plasmacytoid DCs appeared in higher number at week 2. The AhR antagonist CH223191 preferentially augmented the migration of myeloid DCs to the lungs of infected mice.

### Treatment With AhR Ligands Alters the Presence of Innate Lymphoid Cells (ILCs) in the Lungs of *P. brasiliensis* Infected Mice

**Figure 5:**
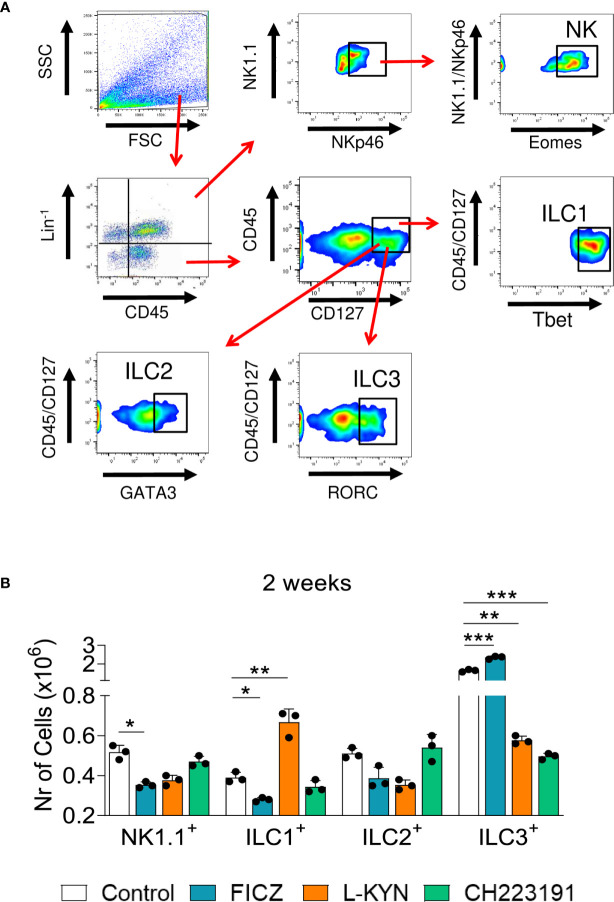
Treatment with AhR ligands alters the expansion and presence of Innate Lymphoid Cells (ILCs) in the lungs of *P. brasiliensis* infected mice. C57BL/6 mice were infected with 1 x 10^6^ *P. brasiliensis* yeasts and treated with FICZ, L-Kyn, CH223191 as previously described. Control mice were treated with PBS. The animals were sacrificed 96 hours and 2 weeks after infection, their lungs removed, macerated, the leukocytes obtained, and analyzed by flow cytometry for ILCs phenotypes (NK 1.1, ILC1, ILC2, and ILC3). **(A)** Gate strategy to define ILCs subsets. Lung leukocytes were first treated with an anti-mouse lineage cocktail (Biolegend) containing antibodies to CD3, Ly6G/Ly6C, CD11b, CD45R/B220, TER 119/erythroid cells, that react with T cells, B cells, monocytes, macrophages, NK cells, and erythrocytes. NK cells were then classified as Lin^+^CD45^+^NK1.1^+^ NKp46^+^Eomes^+^, ILC1 as CD45^+^Lin^-^CD127^+^Tbet^+^, ILC2 as CD45^+^Lin^-^CD127^+^Gata3^+^, and ILC3 as CD45^+^Lin^-^CD127^+^RORC^+^. The cell surface and intracellular markers were measured by flow cytometry and 50.000 cells were counted. **(B)** Number of NK1.1, ILC1, ILC2, and ILC3 positive cells detected in the lungs of mice at 96 hours and 2 weeks after infection. The experiment was repeated twice and data are expressed as M ± SD. *P* values < 0.05 were considered significant (*p < 0.05; **p < 0.005 and ***p < 0.001).

We have also characterized the influence of the AhR ligands on the differentiation of pulmonary ILCs. These cells represent a new family of lymphocytes that do not express receptors for antigens, produce significant amounts of cytokines, and can be cytotoxic when activated. In ILCs, T and B cell receptors are absent, and their development is independent of RAG genes. The different subpopulations of ILCs exhibit transcription factors and cytokines that are prototypical of CD4 + T cell subsets. These characteristics include the shared expression of Tbet and IFN-γ by ILC1 and Th1, GATA-3, IL-5 and IL-13 by Th2 and ILC2; RORC, IL-17, and IL-22 by ILC3 and Th17/Th22 cells, as well as Eomes, IFN-γ and cytotoxic molecules by CD8+ T cells and conventional NK cells (43). **Figure 5A** depicts the gating strategy used to define ILCs subsets. We could demonstrate (**Figure 5B**) that FICZ treatment induced a great expansion of ILC3 but reduced the number of NK1.1 and ILC1 cells. L-Kyn induced the expansion of ILC1 but reduced ILC3. On the other hand, the AhR antagonist CH223191 caused only a profound reduction in the presence of ILC3 lymphocytes in the lungs of *P. brasiliensis* infected mice.

### Treatment With AhR Ligands Alters Cytokine Gene Expression in the Lungs of *P. brasiliensis* Infected Mice

**Figure 6:**
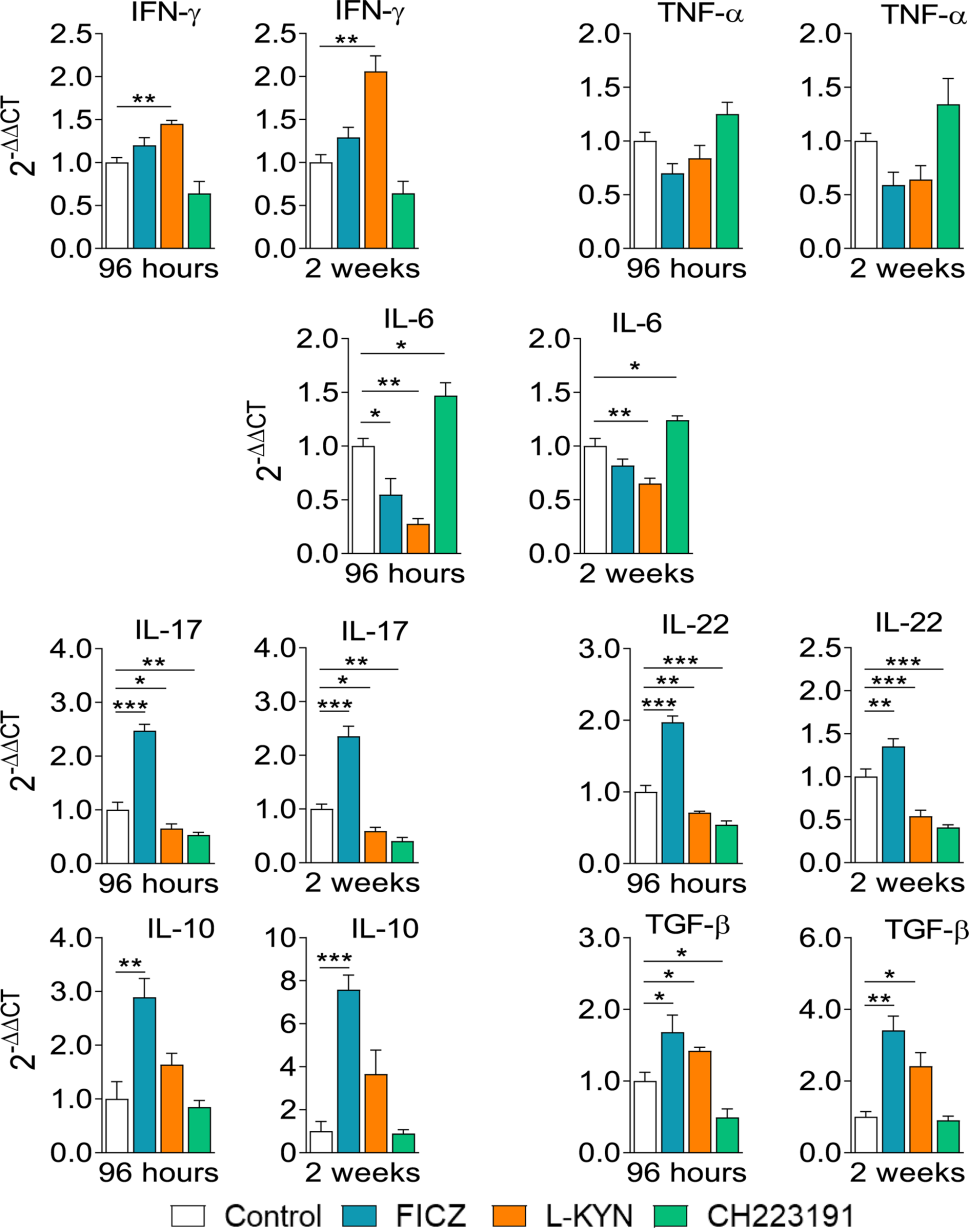
Treatment with AhR ligands alters mRNA expression for pro- and anti-inflammatory cytokines in the lungs of *P. brasiliensis* infected mice. C57BL/6 mice (n = 5) were infected with 1 x 10^6^ *P. brasiliensis* yeasts and treated with FICZ, L-Kyn, CH223191 as previously described. Control mice were treated with PBS. The animals were sacrificed 96 hours and 2 weeks after infection, their lungs removed, macerated, and the RNA obtained analyzed by RT-PCR as described in M&M. The experiment was repeated twice, and data are expressed as M ± SD. *P* values < 0.05 were considered significant (*p < 0.05; **p < 0.005 and ***p < 0.001).

Infected control and AhR ligands treated mice were sacrificed, their lungs removed, macerated, and the relative expression of mRNA for cytokines analyzed by RT-PCR. The results obtained at the two infection periods studied were similar (**Figure 6**). FICZ enhanced the expression of *il-17, il-22, il-10*, and *tgf-b* mRNAs at both periods but reduced *il-6* levels at 96 hours of infection. L-Kyn increased the expression of *ifn-γ *and *tgf-β* but reduced the levels of *il-6*, *il-17*, and *il-22* mRNAs. CH223191, on the other hand, increased the synthesis of *il-6* but reduced *il-17* and *il-22* mRNA at both post-infection periods, but *tgf-β* only at 96 hours after infection.

### Treatment With AhR Ligands Modifies the Levels of Pro- and Anti-inflammatory Cytokines in the Lungs of *P. brasiliensis* Infected Mice

**Figure 7:**
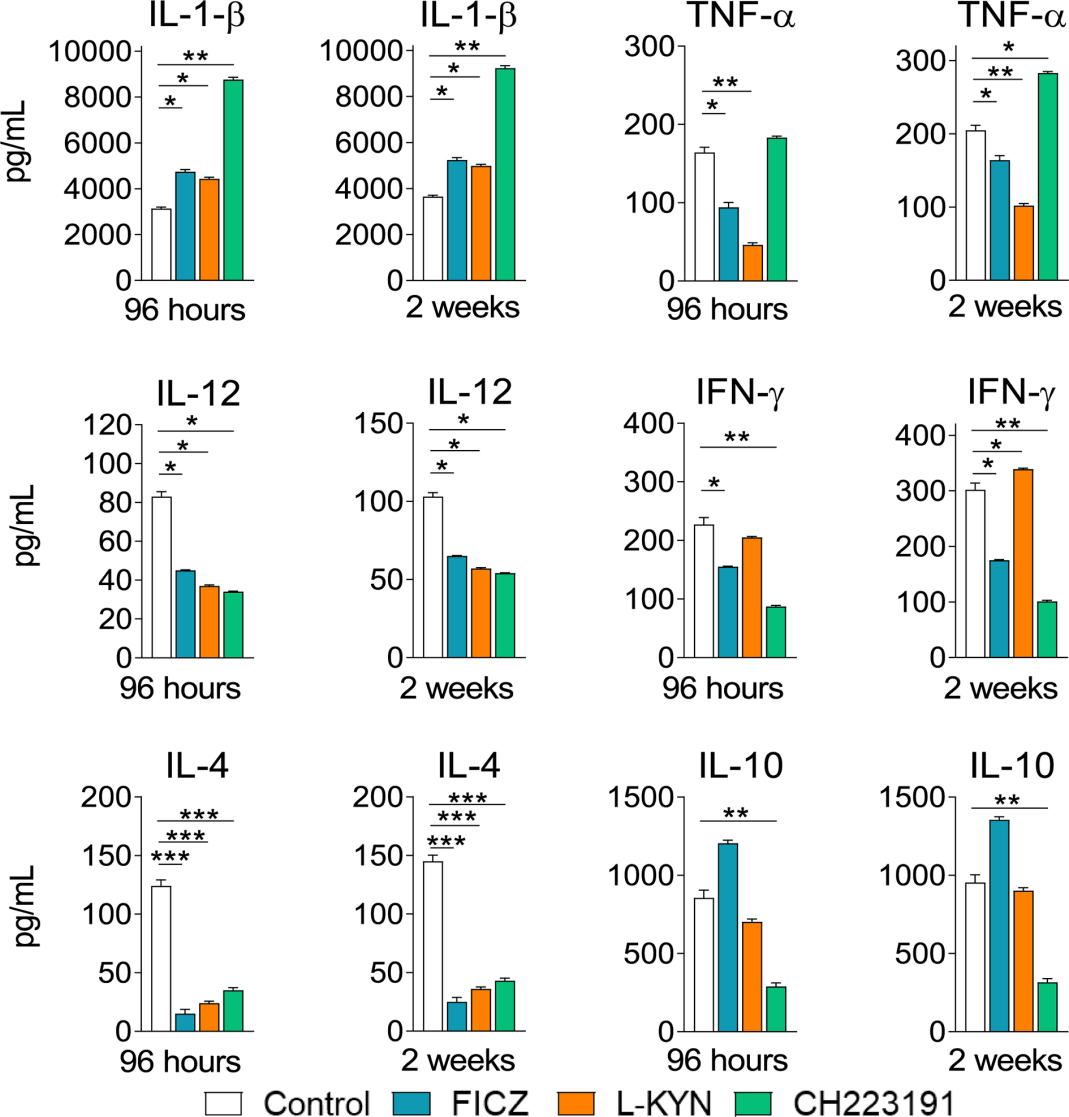
Treatment with AhR ligands increases the levels of pulmonary IL-1β but reduces the levels of cytokines involved in the development or activity of Th1 and Th2 cells. C57BL/6 mice (n = 5) were infected with 1 x 10^6^ *P. brasiliensis* yeasts and treated with FICZ, L-Kyn, CH223191 as previously described. Control mice were treated with PBS. The animals were sacrificed 96 hours and 2 weeks after infection, their lungs removed, macerated and the supernatants analyzed for the presence of cytokines by ELISA. The experiment was repeated twice, and data are expressed as M ± SD. *P* values < 0.05 were considered significant (*p < 0.05; **p < 0.005 and ***p < 0.001).

**Figure 8:**
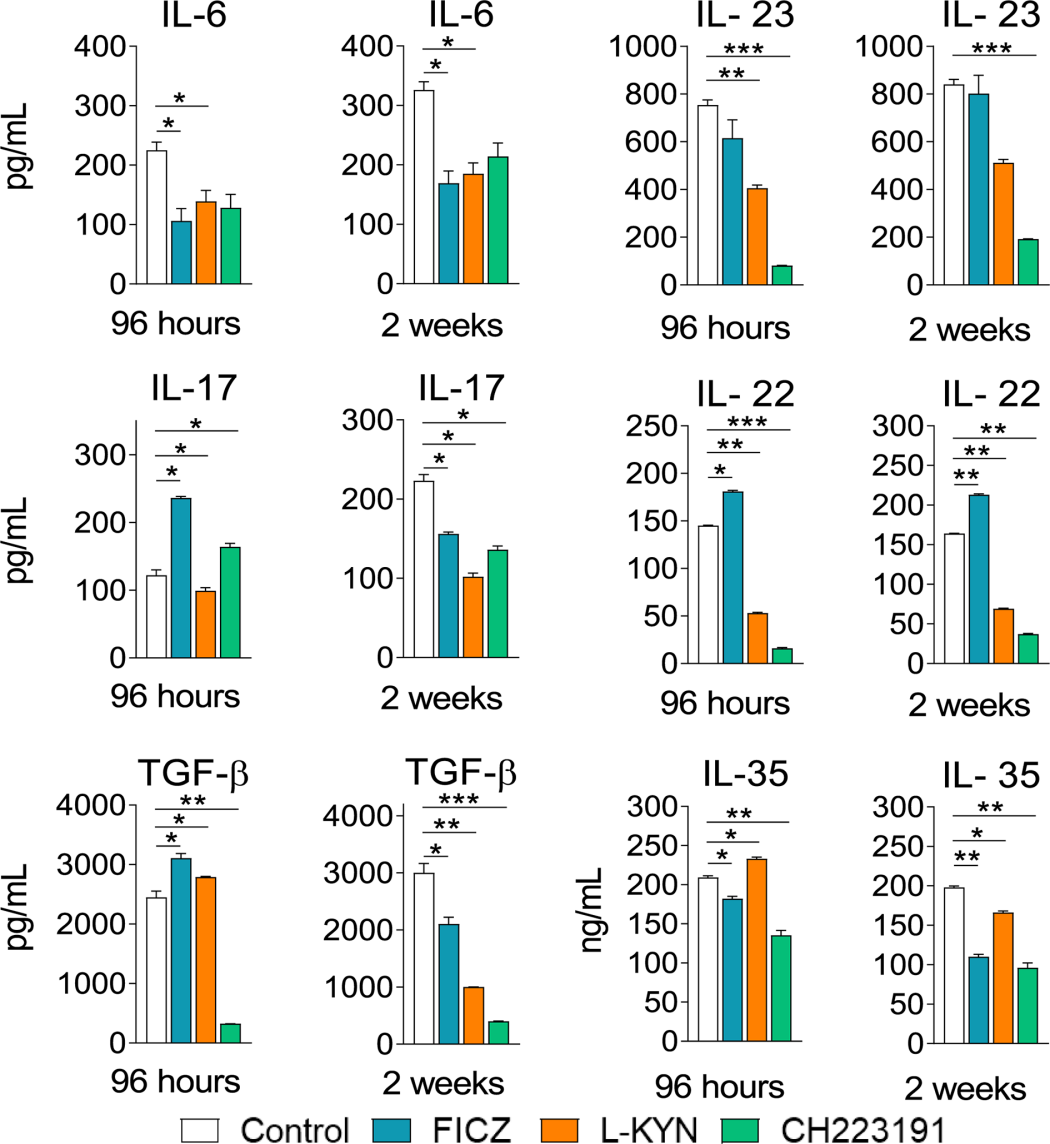
Treatment with AhR ligands alters the levels of pulmonary cytokines involved with Th17 and Treg cells development and activity. C57BL/6 mice (n = 5) were infected with 1 x 10^6^ *P. brasiliensis* yeasts and treated with FICZ, L-Kyn, CH223191, as previously described. Control mice were treated with PBS. The animals were sacrificed 96 hours and 2 weeks after infection, their lungs removed, macerated and the supernatants analyzed for the presence of cytokines by ELISA. The experiment was repeated twice, and data are expressed as M ± SD. *P* values < 0.05 were considered significant (*p < 0.05; **p < 0.005 and ***p < 0.001).

Mice were treated as previously described. Supernatants from lung macerates obtained 96 hours and 2 weeks post-infection were analyzed by ELISA for the presence of pro- and anti-inflammatory cytokines. As can be seen in **Figure 7**, IL-1β was the only cytokine that appeared in increased levels at both periods assayed and treatments used. In contrast, TNF-α and cytokines involved in Th1 (IL-12, IFN-γ) and Th2 (IL-4, IL-10) differentiation or activity appeared in reduced levels in almost all treatments and time points studied. As expected, AhR ligands have also altered the levels of cytokines involved in Th17 and Treg cells differentiation and activity (**Figure 8**). IL-6, IL-23, and TGF-β and were seen in reduced levels at least in one post-infection period after L-Kyn and CH223191 treatments and this was accompanied by reduced levels of IL-17 (week 2) and IL-22 (both infection periods). On the other hand, FICZ increased the levels of IL-17 (96 hours) and IL-22 (96 hours and 2 weeks), whereas CH223191 caused increased IL-17 production only in the first period assayed. Besides, all employed AhR ligands caused at the late time point assayed a reduction in TGF-β and IL-35, two suppressive cytokines involved in Treg cells activity.

### AhR Ligands Modify the Message for AhR, IDO, and Transcription Factors for CD4+ T Cell Subsets

**Figure 9:**
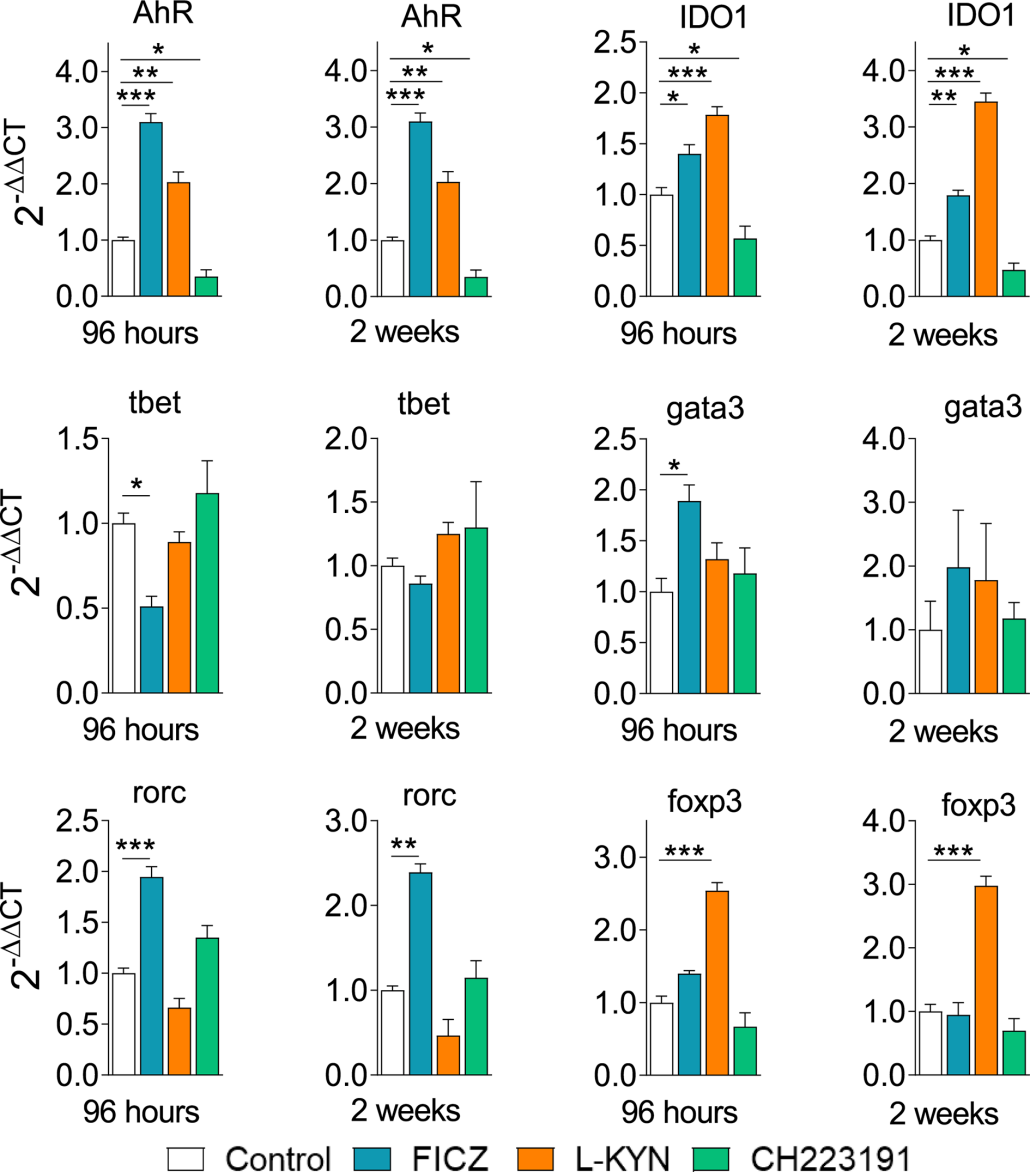
Treatment with AhR ligands alters the expression of mRNA for IDO, AhR, and transcription factors in the lungs of *P. brasiliensis* infected mice. C57BL/6 mice (n = 5) were infected with 1 x 10^6^ *P. brasiliensis* yeasts and treated with FICZ, L-Kyn, CH223191 as previously described. Control mice were treated with PBS. The animals were sacrificed 96 hours and 2 weeks after infection, their lungs removed, macerated, and the RNA obtained was analyzed by RT-PCR as described in M&M. The experiment was repeated twice, and data are expressed as M ± SD. *P* values < 0.05 were considered significant (*p < 0.05; **p < 0.005 and ***p < 0.001).

Control and FICZ, L-Kyn, and CH223191 treated mice were sacrificed, their lungs removed, macerated, and the relative expression of mRNA for AhR, IDO, and master transcription factors for CD4+ T cell subsets differentiation were analyzed by RT-PCR. Similar results were obtained in both periods of infection (**Figure 9**). FICZ and L-Kyn agonists led to increased mRNA expression for IDO and AhR, while the antagonist CH223191 decreased their expression. FICZ reduced *tbet* but increased the expression of *gata3* and *rorc*. L-Kyn treatment caused a robust increase in the *foxp3* message while CH223191 did not significantly change the mRNA levels for all transcription factors assayed.

### AhR Ligands Modify the Expansion of CD4+ T Cell Subsets That Migrate to the Lungs of Infected Mice

**Figure 10:**
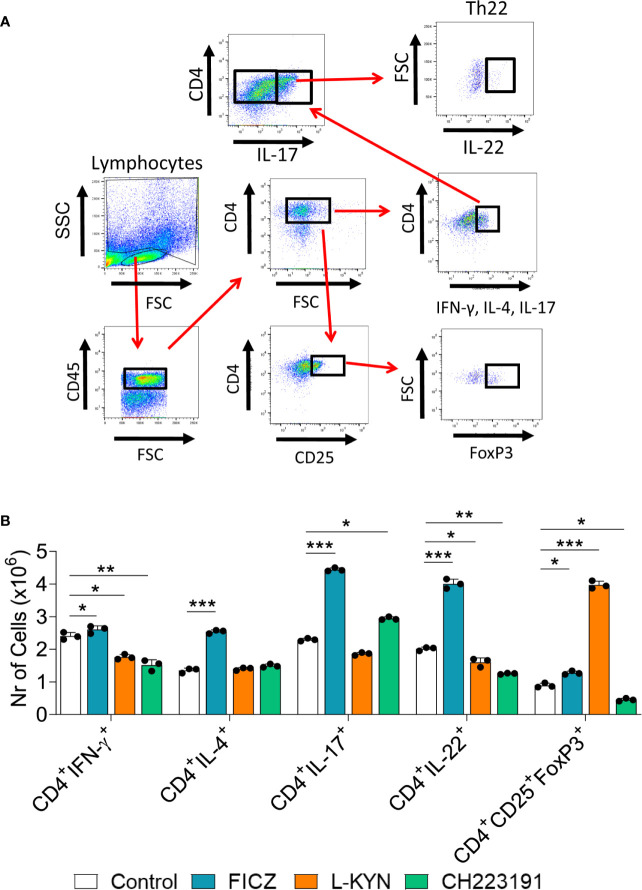
Treatment with AhR ligands alters the differentiation and migration of CD4+ T cell subsets to the lungs of *P. brasiliensis* infected mice. C57BL/6 mice were infected with 1 x 10^6^ *P. brasiliensis* yeasts and treated on alternate days with FICZ, L-Kyn, or CH223191 at doses of 200, 800, or 400 μg/animal, respectively. Control mice were treated with PBS. The animals were sacrificed 2 weeks after infection, their lungs removed, macerated and the CD4+ T lymphocytes evaluated by flow cytometry for intracellular expression of Th signature cytokines (IFN-γ, IL-4, IL-17, IL-22) and the Treg phenotype (CD4+CD25+Foxp3+). **(A)** Gate strategy to define T cell subsets. **(B)** Number of Th1, Th2, Th17, Th22, and Treg cells present in the lungs of control and AhR ligands treated mice. The experiment was repeated twice, and data are expressed as M ± SD. *P* values < 0.05 were considered significant (*p < 0.05; **p < 0.005 and ***p < 0.001).

Mice were treated as previously described. Two weeks after infection, isolated lung leukocytes were analyzed by flow cytometry regarding the presence of CD4+ T cell subsets. The gating strategies used to define CD4+ T cell subsets are shown in **Figures 10A, B** depicts the number of these cells present in the lungs of treated and untreated mice. The FICZ agonist significantly increased the migration of all cell T cell subpopulations (Th1, Th2, Th17, Th22, and Treg). Concomitant with the elevated expression of foxp3 mRNA, L-Kyn treatment caused a vigorous increase in Treg cells associated with Th1 and Th22 decrease. The CH223191 antagonist, on the other hand, reduced the migration of Th1, Th22, and Tregs but increased the presence of pulmonary Th17 lymphocytes. A general view of these results suggests that FICZ has a great inducing effect on the differentiation and migration of Th2, Th17, and Th22 subpopulations. L-Kyn has a great inducing effect on Treg cells, while the greatest effect of CH223191 is the expansion of Th17 lymphocytes associated with concomitant reduction of Treg cells.

## Discussion

Traditionally considered a mediator in the toxic response to dioxin, AhR was later described as an important regulator of the immune response, including the immunity against infectious agents (13). In addition to inducing detoxifying enzymes, AhR modulates the differentiation and activity of innate and adaptive immune cells (10, 11, 13, 44), profoundly influencing the outcome of infectious and inflammatory processes (13, 45).

In pulmonary PCM, our group showed that *P. brasiliensis* infection induces and activates the enzyme IDO that causes TRP deprivation and L-Kyn production. TRP shortage leads to reduced fungal growth and diminished infection of DCs and macrophages. The enhanced L-Kyn synthesis, *via* AhR activation, increases the expansion of Treg cells that control excessive Th17-mediated tissue pathology (34–37, 46). Our studies also demonstrated an important interconnection between IDO and AhR expression, where a balanced activation of these mediators proved to be fundamental for the control of fungal immunity and disease tolerance (34–37, 46).

Since its description, the immunomodulatory effect of AhR has been associated with the control of both, exacerbated and deficient immune responses. These opposed effects result from the diverse interactions between AhR and its ligands and environmental cytokines, among other factors (10, 11, 47–49). Thus, the AhR activation can enhance pro-inflammatory responses, usually mediated by the Th1/Th17 subpopulations, or anti-inflammatory or suppressive immunity mediated by Treg and Tr1 cells (10, 11, 14). Environmental cytokines at the time of cell differentiation are fundamental to this process, and each pathogen and each specific disease induces complex patterns of mediators that are greatly influenced by the host’s genetic pattern, as well as more general environmental and metabolic factors (13, 14, 45). Indeed, comparing the effect of four different AhR agonists in the immunity and severity of a viral infection, Boule et al. (45) elegantly demonstrated that ligand metabolism and binding affinity, but not the chemical source, determines their immunological effects. Despite this great variability, some studies have shown that AhR agonists that are more difficult to be metabolized (eg: TCDD) induce increased expression of Treg (or Tr1) cells, while others, such as FICZ, induce greater polarization of T cells to the Th17 phenotype that synthesizes IL-17 and are also competent IL-22 producers (41, 50, 51). Th22 subpopulations, on the other hand, are most dependent on AhR expression (52, 53).

Our previous findings demonstrating the important role of the IDO/AhR/Treg/Th17 axis in the control of pulmonary PCM led us to comparatively investigate the immunomodulatory effect of two different agonists and one antagonist of AhR signaling. Thus, different groups of infected mice were treated with FICZ, a high-affinity agonist, L-Kyn, a low-affinity agonist, and CH223191 a low-affinity antagonist of AhR (49). The findings here reported demonstrate the peculiar effects of each of the AhR ligands studied in the control of pulmonary PCM. Some similar effects were shared by the FICZ and L-Kyn agonists, such as reduced fungal loads, decreased number of pulmonary CD11c+ myeloid cells expressing activation markers (IA, CD40, CD80, CD86), and increased expression of AhR and IDO. In contrast, these effects were opposed when the animals were treated with the AhR antagonist CH22319: there was an increase in pulmonary fungal loads and the number of CD11c+ leukocytes expressing activation markers, besides a drastic reduction in the expression of AhR and IDO. In the adaptive immune response, however, each of these ligands induced a characteristic profile. The large expansion of myeloid, lymphoid, and plasmacytoid DCs induced by FICZ increased all CD4+ T cell subpopulations (Th1, Th2, Th17, Th22, and Treg), but with the predominance of the Th17 and Th22 subsets. L-Kyn, which preferentially activated plasmacytoid DCs, possibly tolerogenic (35, 46, 54, 55), reduced the expansion of Th1 and Th22 cells but caused a great expansion of Treg cells differentiation. CH223191, the AhR antagonist, induced the preferential expansion of myeloid DCs, reduced the number of Th1, Th22, and Treg lymphocytes, but caused a significant increase in Th17 cells. This compound also induced a great decrease in the synthesis of pulmonary cytokines, with emphasis on the reduction of the various cytokines associated with the suppressive function of Tregs (IL-10, IL-35, and TGF-β). ILC3 was the most ILC subpopulation affected by AhR ligands treatment. While treatment with FICZ promoted a large increase in ILC3, the CH223191 antagonist drastically reduced this cell population. L-Kyn increased the number of ILC1 in the lungs but, similarly to CH223191, reduced the expansion of ILC3.

Treatment with FICZ augmented the number of CD11c+ myeloid cells expressing some intracellular pro- and anti-inflammatory cytokines that appear to have influenced the differentiation of all CD4+ T cell subsets here described. Interestingly, FICZ treatment caused a significant reduction in almost all secreted pulmonary cytokines except for IL-1β, TGF-β, IL-17, and IL-22, all cytokines involved in Th17 expansion and activity, and a prominent Th cell expanded by this treatment. Besides, the characterization of lung mRNA demonstrated constant increases in *ahr*, *rorc*, *il-22*, and *il-17*, indicating the tendency of this AhR agonist to induce prevalent Th17 and Th22 responses. This finding is in agreement with a pioneering publication by Quintana et al. (11) demonstrating that FICZ induces the production of transcription factors and cytokines that coordinate the preferential differentiation of T cells to the Th17 profile. The increased differentiation of Th22 is also in agreement with the IL-22 dependence of AhR expression (56). This AhR agonist also induced a large increase in ILC3 lymphocytes, IL-17, and IL-22 producers and highly dependent on the transcription factors RORc and AhR [12, 57).

The analysis of mRNA present in the lungs of L-Kyn treated mice confirmed a large increase in the message for AhR and IDO in the two post-infection periods analyzed. Analogous to FICZ, treatment with L-Kyn increased the numbers of CD11c+ myeloid cells expressing pro- and anti-inflammatory cytokines. However, a large expansion of plasmacytoid DCs, which have a tolerogenic profile in pulmonary PCM (35, 46), was detected in L-Kyn treated mice. Indeed, L-Kyn treatment induced a robust increase in pulmonary Treg (CD4+CD25+Foxp3+) cells, and this finding was associated with the large expression of mRNA for Foxp3 in the two periods of infection studied. The profile of soluble pulmonary cytokines in L-Kyn treated mice showed a consistent reduction in almost all cytokines assayed, except for TGF-β and IL-35 that could be associated with the increased Treg cells expansion here described. Still, a reduction in Th22 cells observed at the second week post-infection, and this finding was accompanied by a reduction in IL-22 in the lung supernatants and in mRNA for IL-22 of L-Kyn treated mice at the two periods of disease assayed. Since the synthesis of IL-22 is highly dependent on AhR (56), and this transcription factor appeared at high levels as protein in myeloid cells, and as mRNA in total lung cells, we can suppose that the reduction in Th22 lymphocytes mediated by L-Kyn treatment could have been influenced by the concomitant activity of other transcription factors or the increased expansion of Treg cells. In this respect, it is also worth mentioning the great reduction of ILC3, which depends on the RORc transcription factor for IL-17 synthesis, but on AhR for IL-22 production (12, 57). The decrease in ILC3 was consistently accompanied by a reduction in IL-17 and IL-22 in the lung supernatants, but not in the expression of AhR. However, to better analyze the effect of AhR on ILCs, we should have phenotypically characterized the simultaneous synthesis of IL-17 and IL-22 by ILC3 as well as the NCR-IL-22 subpopulation whose IL-22 synthesis is AhR-dependent (57). In summary, treatment with L-Kyn appears to have exerted a predominant anti-inflammatory effect on PCM, mainly due to the early increased expression of AhR and IDO that controlled the fungal load and established a low expression of activation molecules by myeloid cells, a predominant expansion of plasmacytoid DCs and an increased differentiation of Foxp3+ Treg cells.

The AhR antagonist CH223191, as expected, caused opposed effects to the studied agonists, in particular FICZ. CH223191 treatment reduced the number of IDO+ CD11c+ cells and increased the number of viable *P. brasiliensis* yeasts present in the lungs at both time points studied. This agrees with our previous reports demonstrating the increased *P. brasiliensis* growth when IDO is metabolically inhibited or genetically ablated, possibly by the increased TRP availability for fungal metabolism (34–37), The reduction in IDO was concomitant with that of AhR, the two components that are mutually controlled (14). Contrasting the treatments with FICZ and L-Kyn, CH223191 caused a large increase in the number of CD11c+ myeloid cells expressing activation molecules, intracellular TNF-α and a preferential expansion of myeloid DCs. It was also observed a reduction in Th1, Th22, and Treg cells besides an increase in Th17 cells. The reduction in Treg cells was concomitant with decreased levels of several cytokines (IL-10, TGF-β, and IL-35) associated with their anti-inflammatory function. The reduction of Th1 lymphocytes was concomitant with a low presence of IFN-γ in pulmonary cell supernatants, while the increase in Th17 lymphocytes occurred with an increase in IL-17 only at 96 hours after infection. Contrasting the AhR agonists, CH223191 reduced, as expected, the mRNA expression for IDO and AhR. This reduced AhR expression was associated with decreased numbers of AhR-dependent ILC3 since we did not notice a reduction in RORc expression. As a whole, treatment with an AhR antagonist reproduced the main findings that we observed in *P. brasiliensis* infected AhR^-/-^ mice (37), increased fungal loads, and Th17 immunity not adequately controlled by insufficient Treg cells expansion.

The lung is a barrier organ that expresses AhR at high levels (58). This transcription factor is expressed by epithelial and immune cells, is involved in mucous secretion (59) and the balanced immune response in the lung (49). In accordance, our studies have demonstrated the important participation of AhR in the control of disease severity and immune response in pulmonary PCM (34–37, 46). The present study allowed us to demonstrate that this fungal infection can be modulated by AhR ligands in opposed directions as previously demonstrated in other experimental models (10, 11, 45), suggesting their therapeutic use in different forms of the disease. FICZ, due to its fungicidal effect and prominent pro-inflammatory activity that mediates the increased expansion of all T cell subsets but prevalent Th17 differentiation, could be used as adjunct therapy of severe human PCM characterized by T cell anergy and high fungal loads (25). L-Kyn, which favors the expansion of Treg cells but reduces fungal loads, could be used as an immunomodulator in those situations of severe tissue damage associated with hyperreactivity of the immune system, which occasionally occurs in the human PCM (60). The use of the AhR antagonist, which leads to excessive fungal growth and increased Th17 immunity should be therapeutically discarded since its effect mimics those observed with AhR^-/-^ mice, where the uncontrolled fungal growth associated with unrestrained pro-inflammatory reactions lead to extremely severe disease. Finally, our data encourage further studies on the immunomodulation of PCM by AhR agonists and open the perspective of their use in future immunotherapeutic procedures.

## Data Availability Statement

The original contributions presented in the study are included in the article/**Supplementary Material**. Further inquiries can be directed to the corresponding author.

## Ethics Statement 

The animal study was reviewed and approved by Committee on Animal Experiments of the Institute of Biomedical Sciences of the University of São Paulo (Proc.180/11/CEEA).

## Author Contributions

EA and VC conceived and planned experiments. EA, NP, CF, NG, and TC carried out the experiments. EA, FL, and VC contributed to the interpretation of the results. EA, FL, and VC wrote the paper. EA, NP, and FL prepared the figures. VC supervised the project and provided financial support. All authors contributed to the article and approved the submitted version.

## Funding

This work was supported by a grant from the Fundação de Amparo à Pesquisa do Estado de São Paulo (FAPESP- grant to VC 2011/51258-2 and 2016/23189-0; fellowship to EA 2014/18668-2; grant to FL 2018/14762-3; fellowship to NP 2019-09278-8), and Conselho Nacional de Pesquisa (CNPq).

## Conflict of Interest

The authors declare that the research was conducted in the absence of any commercial or financial relationships that could be construed as a potential conflict of interest.
