# Supplementary material for: AhR Ligands Modulate the Differentiation of Innate Lymphoid Cells and T Helper Cell Subsets That Control the Severity of a Pulmonary Fungal Infection
Source: Front Immunol. 2021 Apr 16;12:630938. doi: 10.3389/fimmu.2021.630938 (PMC8085362; doi:10.3389/fimmu.2021.630938)
Supplement: Supplementary file 1 [file DataSheet_1.pdf]

Supplementary Table-1 List of monoclonal antibodies used in flow cytometry studies

Primary antibodies	Fluorocrome	Suplier	Reference
CD45	BV510	Biolegend	30-F11
CD45	BV421	BD Bioscience	30-F11
CD45	FITC	Biolegend	30-F11
CD45	PE Cy7	Biolegend	30-F11
CD45	PE Cy5	Biolegend	30-F11
CD11b	BV421	Biolegend	M1/70
CD11b	APC Cy7	BD Bioscience	M1/70
F4/80	APC	Biolegend	BM8
F4/80	Percp 5.5	Biolegend	BM8
CD11c	BV421	Biolegend	N418
CD11c	PE Cy7	BD Bioscience	HL3
CD11c	APC	eBioscience	N418
CD80	Percp 5.5	BD Bioscience	16.10A1
CD86	PE	BD Bioscience	GL1
CD40	FITC	BD Bioscience	3.23
MHC II	APC Cy7	Biolegend	M5/114.15.2
CD4	FITC	BD Bioscience	GK1.5
CD4	APC Cy7	Biolegend	GK1.5
CD8	APC Cy7	Biolegend	53-6.7
IL-4	PE Cy7	BD Bioscience	11B11
IL-10	PercP 5.5	eBioscience	VC10-4B9
IL-12	FITC	BD Bioscience	C15.6
IL-17	BV510	BD Bioscience	TC11-18H10
IL-22	APC	Biolegend	Poly5164
IL-22	PercP 5.5	Biolegend	Poly5165
IL-6	APC	BD Bioscience	MP5-20F3
IL1-β	eFluor780	eBioscience	NJTEN3
TNF-α	PE Cy7	BD Bioscience	MP6-XT22
TGF-β	PE Cy7	R&D	1D11
FoxP3	Alexa Fluor 488	BD Bioscience	MF23
FoxP3	PE	eBioscience	NRRF-30
GATA3	Alexa Fluor 488	Biolegend	16E10A23
RORc	APC	eBioscience	B2D
IFN-γ	BV421	Biolegend	XGM1.2
Tbet	PE	Biolegend	4B10
AhR	PE	eBioscience	4MEJJ
AhR	eFluor660	eBioscience	FF3399
CD25	eFluor 450	eBioscience	eBio3C7
CTLA-4	BV421?	Biolegend	VC10-4B9
IDO-1	FITC	R&D	700838
Lineage cocktail	BV421	Biolegend	17A2; RB6-8C5; RA3-6B2; Ter-119; M1/70
CD127	BV510	Biolegend	A7.R34
NK1.1	BV510	Biolegend	PK136
NPk46	PE Cy7	Biolegend	29A1.4
Eomes	PE	eBioscience	Dan11mag
CD317	PE	Biolegend	129C1
CD16/32	APC	Biolegend	93
